# Mortality Rates in People With Convulsive Epilepsy in Rural Northeast China

**DOI:** 10.3389/fneur.2020.01013

**Published:** 2020-09-11

**Authors:** Jing Li, Xinyue Zhang, Nan Li, Danyang Zhao, Guangjian Li, Weihong Lin

**Affiliations:** Department of Neurology, The First Hospital of Jilin University, Changchun, China

**Keywords:** phenobarbital (PB), sodium valerate, mortality, convulsive epilepsy, northeast China

## Abstract

**Purpose:** To examine the mortality rate and causes of death of phenobarbital (PB) monotherapy and sodium valproate (VPA) monotherapy in patients with convulsive epilepsy in rural northeast China and compare the differences in the results between the two antiepileptic drugs.

**Methods:** Patients with convulsive epilepsy were recruited by trained public health workers in a project for epilepsy prevention and treatment. Patients were enrolled between January 2010 and December 2018 and were treated with PB or VPA. Mortality rate (MR), the proportional mortality ratio (PMR) for each cause, standardized mortality ratio, and years of potential life lost (YPLL) for sex, age, and cause were estimated based on the 2018 Chinese rural population.

**Results:** A total of 3,916 patients with convulsive epilepsy enrolled in the study, of whom 3,418 received PB and 498 received VPA. There were 325 reported deaths (300 from the PB group) during the follow-up period. The MRs were 9.96 and 5.73% in the PB and VPA groups, respectively. The overall SMRs were 12.92 (95% confidence intervals [CI]: 11.50–13.93) and 7.39 (95% CI: 4.78–10.91), for the PB and VPA groups, respectively. Cerebrovascular disease and heart disease were the major causes of death in both treatment groups. The average YPLL for the PB group (21.9 years) was higher than that for the VPA group (13.4 years).

**Conclusion:** This is the first epidemiological study to examine the MR of patients with epilepsy in rural northeast China. Our study is somewhat different from previous studies reported in China, and we provide new relevant data from northeast China.

## Introduction

Epilepsy is a common chronic disease of the brain that affects people of all ages. There are an estimated 65 million people with epilepsy (PWE) worldwide, with roughly 80% of them living in developing countries (1, 2). Mortality among PWE in low- and middle-income countries (LMICs) is higher than that in high-income countries (3), and it is also estimated to be 2.6-fold higher than in general populations of LMICs (4). Treatment gap may be one of the reasons contributing to the higher mortality rate (MR). The causes of the epilepsy treatment gap in developing countries have been related to the health systems, mainly regarding inadequate skilled workforce, cost of treatment, and unavailability of drugs (5). Statistically, three-quarters of PWE in LMICs do not receive the treatments they need (6). Phenobarbital and sodium valproate are two traditional broad-spectrum antiepileptic drugs (AEDs) that are inexpensive, efficacious, and usually taken with good compliance.

To study the epilepsy treatment gap in rural areas, an epilepsy management program, which treats people with convulsive epilepsy with phenobarbital or sodium valproate for free, was initiated in rural China with support from the Mental Health Division of the World Health Organization as well as the Bureau of Disease Prevention and Control of the National Health Commission of China in 2000 (7). Between 2006 and 2013, premature mortality was reported among PWE who were treated with phenobarbital in rural areas of China (8–10). While these studies mainly covered midland and southern areas, these studies had the longest observation periods of up to 6.4 years.

To our knowledge, there have been no reports describing premature mortality and long-term observations in the rural areas of northeast China. In this study, we provide data regarding the MR and causes of death of people with convulsive epilepsy who were treated with phenobarbital or sodium valproate as a monotherapy in an unstudied area in China.

## Materials and Methods

### Patients

The study was approved by the Ethics Committee of the First Hospital of Jilin University, and all patients or their guardians (for patients under 16 years of age) provided written informed consent for participation.

From 2010 to 2018, seven counties in the Jilin province of China joined the project for epilepsy prevention and treatment. These areas covered a population of 3,112,100 people, from whom we recruited patients diagnosed with convulsive epilepsy by public health workers who had received basic training in the diagnosis and management of epilepsy. The diagnostic criteria for convulsive epilepsy were as follows: patients who displayed two of the following symptoms that included loss of consciousness, rigidity, or generalized convulsive movements; and at least one of the following symptoms that included urinary incontinence, bitten tongue or an injury sustained in a fall, post-seizure fatigue, or headache or muscle aches after seizure (11). These patients were eligible to be recruited in the study.

PWE who met the following criteria were treated with phenobarbital or sodium valproate monotherapy: at least two seizures within one year before the investigation, or at least two unprovoked (or reflex) seizures occurring >24 h apart; convulsive seizures in addition to other types of seizures; and no previous treatment or irregular treatment with phenobarbital or sodium valproate.

The exclusion criteria for the phenobarbital monotherapy group were as follows: patients who experienced seizures during pregnancy only, seizures associated only with alcohol or drug reduction, patients aged <2 years or weighing <10 kg, history of ADHD, phenobarbital allergy, progressive neurological diseases, history of epileptic status, or patients receiving regular effective treatment with an antiepileptic drug (12).

Patients with any of the following characteristics were not eligible for the treatment of sodium valproate: a history or family history of drug-induced jaundice, liver disease or significant liver damage, blood diseases, renal impairment, hypertension, active mental illness, history of allergies to valproic acid-based AEDs, age <4 years, progressive neurological disorders, ongoing treatment with other AEDs, or history of poor compliance (13).

### Follow-Up Survey and Causes of Death

**Figure 1:**
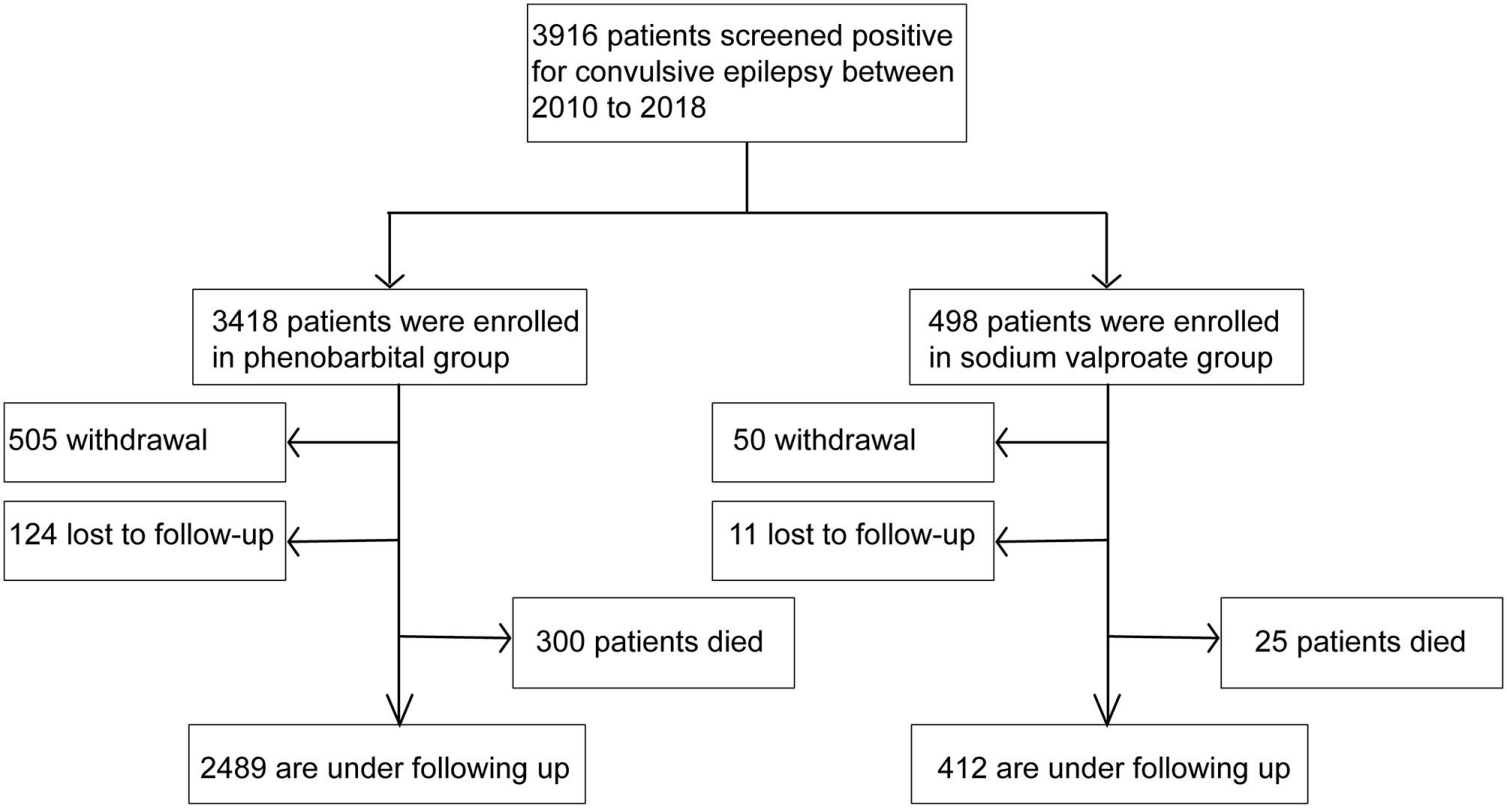
Follow-up survey and causes of death.

Follow-up surveys were conducted once a month on average by the public health workers at the village level. Data on basic demographics, seizure frequency, the dose of drugs prescribed, and adverse reactions were collected in the follow-up survey. Demographics, the date, and cause of death of any patient with an accident or death were recorded in detail by the public health workers (Figure 1). The cause of death was based on that listed on the death certificates and verbal autopsies (14), which were conducted by expert clinicians using the information on cause of death ascertained through interviews with families or neighbors of the deceased. The cause of death was classified according to the 10th Edition of the International Classification of Diseases (ICD-10).

Sudden unexpected death in epilepsy (SUDEP) (15) was defined as sudden, unexpected, witnessed or unwitnessed, non-traumatic, and non-drowning death in patients with epilepsy, with or without evidence of a seizure and excluding documented status epilepticus, in which a toxicologic or anatomic cause of death could not be determined in a postmortem examination. Similar to the definition of definite SUDEP, but without postmortem and toxicologic data, death can be categorized as probable SUDEP (16).

### Statistical Analysis

Person-years at risk were computed for each subject from the date of study entry until the date of death, or the end of follow-up for those who survived. MR was calculated as the number of deaths during follow-up divided by the total number in the study cohort (17). The proportional mortality ratio (PMR) was calculated as the proportion of deaths due to a specific cause in those who died (17). Standardized mortality ratio (SMR), the ratio of the observed number of deaths in the study group divided by the expected number of deaths, was estimated based on age-specific, gender-specific, and cause-specific MRs in the Chinese population in 2018 (18), and the results were given with 95% confidence intervals (CIs). Years of potential life lost (YPLL), a metric we adopted for both men and women, was calculated by subtracting an individual's age at the time of death from the age of 75 years. The estimated number of YPLL for each subject was then summed up to obtain the total YPLL. YPLL for a specific cause of death was also calculated. A survival (Kaplan–Meier) analysis was often used to visually summarize time-to-event data, and it was used to estimate the difference between the two treatment groups.

Values for continuous variables are expressed as the mean ± standard deviation (SD), and values for categorical variables are expressed as frequencies (%). Student's *t*-test, analysis of variance (ANOVA), Pearson's Chi-squared test, the rank-sum test, and Fisher's exact test were used to compare the continuous variables and categorical variables. All *p*-values were estimated in a two-tailed manner. Differences with a *p* < 0.05 were considered statistically significant. The data were analyzed using SPSS for Windows, Version 16.0 (SPSS Inc., Chicago, IL, USA).

## Results

According to the admission criteria, a total of 3,916 patients with convulsive epilepsy were enrolled in this study between January 2010 and December 2018, of whom 3,418 patients (1947 men) were treated with phenobarbital (PB group) and 498 patients (294 men) were treated with sodium valproate (VPA group). During the follow-up period, 135 were lost to follow-up (124 from the PB group) and 325 died (300 from the PB group). Demographic information of the study cohort and the deceased patients are detailed in Table 1.

**Table 1:** Demographic characteristics of the patients with convulsive epilepsy.

	**Phenobarbital**		**Sodium valproate**	
	**All**	**Death**	**All**	**Death**
Number	3,418	300	498	25
Gender, *N* (%) Male	1,947 (56.96)	182 (60.67)	294 (59)	16 (64)
Female	1,471 (43.04)	118 (39.33)	204 (41)	9 (36)
Age, years (SD)	41.79 (15.42)	52.85 (16.95)	44.09 (16.13)	62 (16.54)
Age group, *N* (%) ≤17 years	233 (6.8%)	6 (2)	29 (5.82)	0
18–40 years	1,257 (36.78%)	57 (19)	172 (34.54)	2 (8)
41–65 years	1,745 (51.05%)	164 (54.67)	247 (49.60)	9 (36)
≥66 years	183 (5.35%)	73 (24.33)	50 (10.04)	14 (56)
Onset age, years (SD)	23.08 (17.01)	31.06 (21.33)	29.29 (21.32)	51.32 (22.24)
Duration, years (SD)	18.75 (13.83)	21.90 (15.64)	15.50 (14.27)	10.68 (14.34)
Follow-up time, years (SD)	5.39 (2.09)	2.81 (1.98)	2.23 (1.58)	1.98 (1.27)

*SD, standard deviation*.

**Figure 2:**
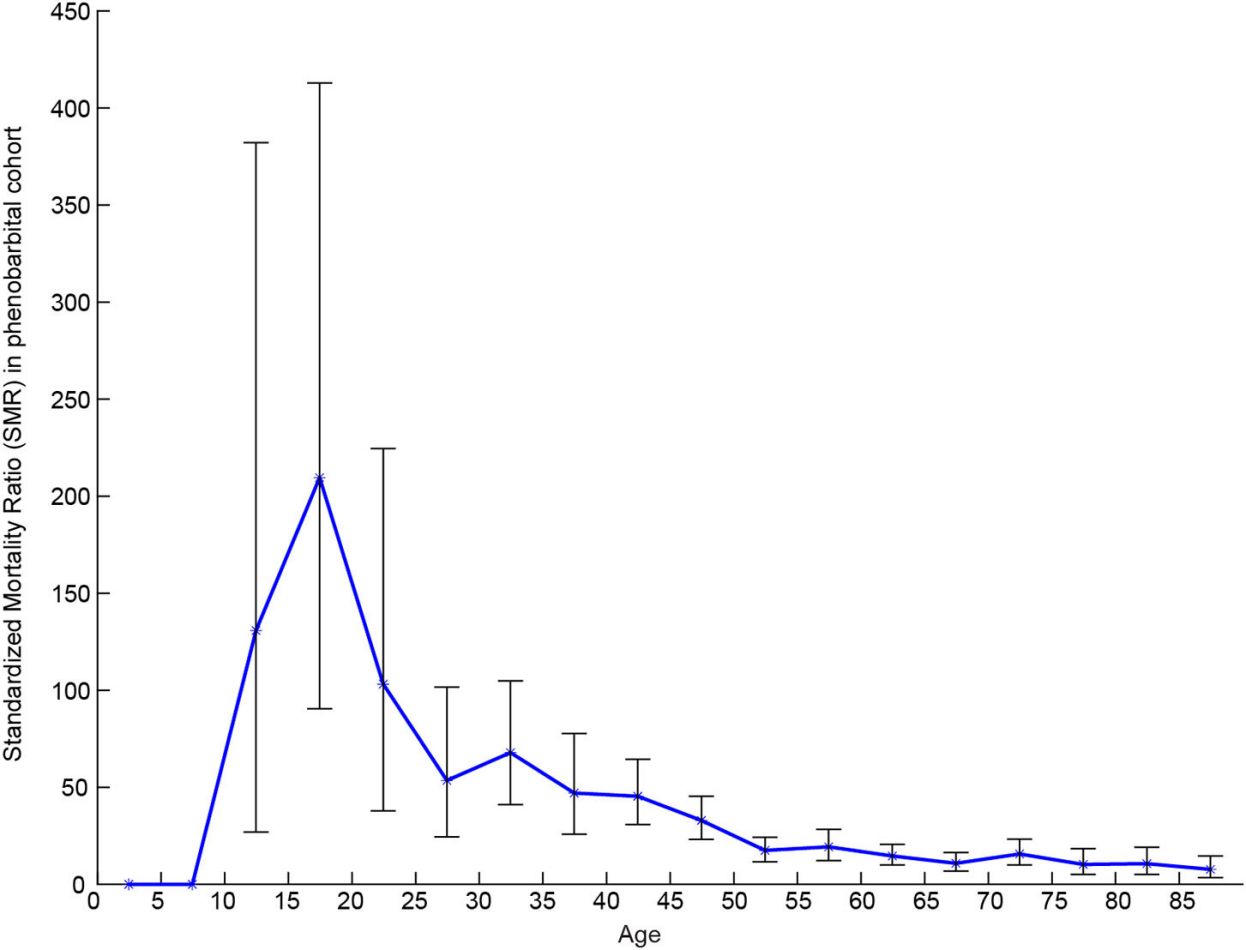
SMR of the PB group.

**Figure 3:**
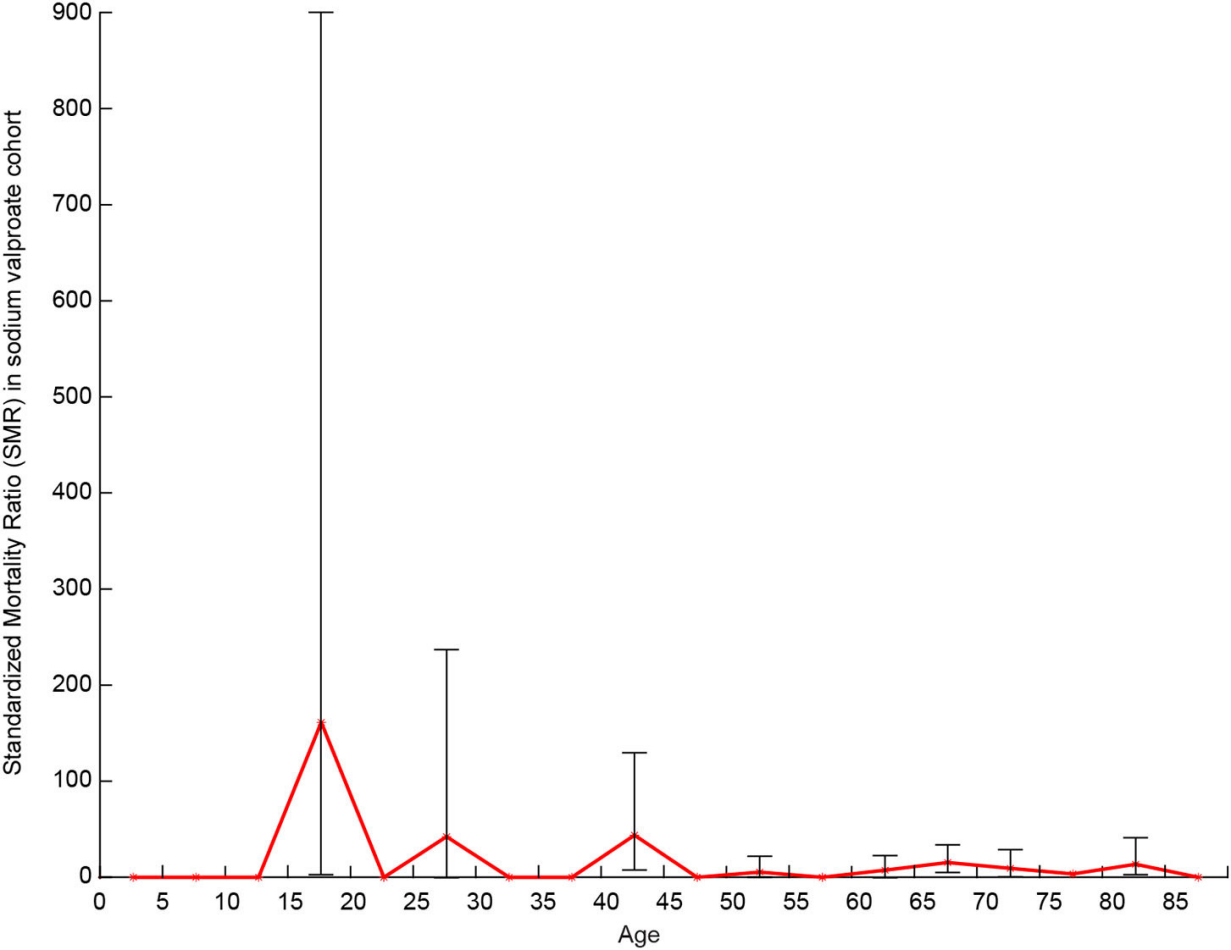
SMR of the VPA group.

**Figure 4:**
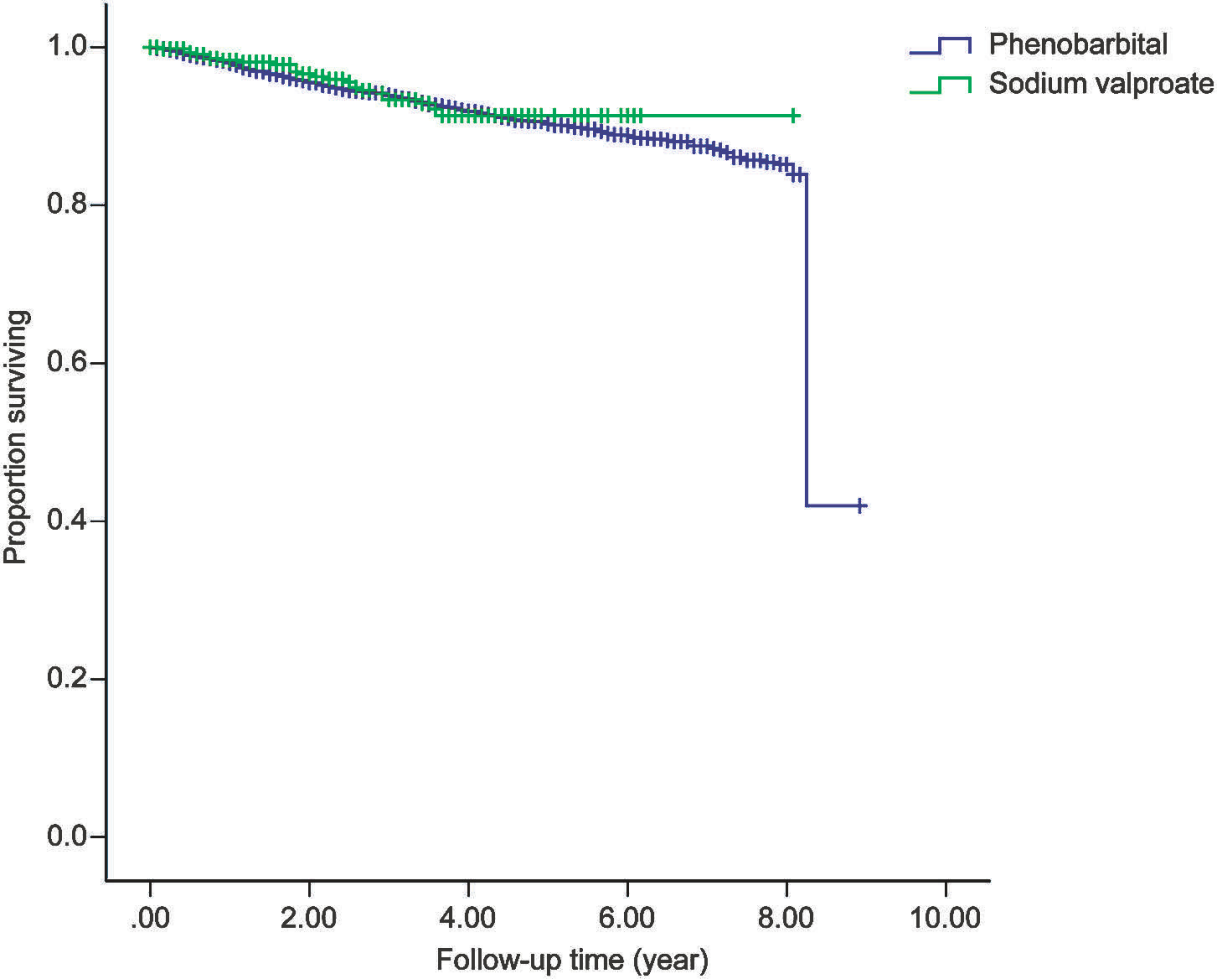
Survival analysis for the two treatment groups in a conventional Kaplan-Meier graphical display.

The MR was 9.96% in the PB group (mean follow-up time, 5.39 years) and 5.73% in VPA group (mean follow-up time, 2.23 years). The SMRs of the PB group were 12.17 (95% CI: 10.46–13.41) for men and 14.06 (95% CI: 11.64–15.87) for women. In the VPA group, the SMRs were 7.08 (95% CI: 4.04–9.94) for men and 7.73 (95% CI: 3.54–12.21) for women. The mean YPLL for men in the PB group was higher than that of women (23.24 vs. 20.47 years, respectively). In contrast, in the VPA group, the mean YPLL of men was lower than that of women (11.88 vs. 15 years, respectively). Figure 2 shows that the SMR of the PB group increased in all age groups and was especially high (between 50 and 210) in patients under 35 years of age. Figure 3 shows that the SMR of the VPA group were highest in patients 15–19 years of age (161.30; 95% CI: 4.08–898.70). Figure 4 shows the survival analysis for the two treatment groups in a conventional Kaplan–Meier graphical display. There was no significant difference between the two groups (*p* > 0.05).

Cause-specific PMRs and SMRs are shown in Table 2. Cerebrovascular disease (32 and 40% of the deceased patients in the PB and VPA groups, respectively) and heart disease (28 and 16% of the patients in the PB and VPA groups, respectively) were the major causes of death in both treatment groups. In the PB group, this was followed by death due to a tumor (7.3%), lung diseases (4%), status epilepticus (4%), and accidental causes including drowning (0.7%), injury (0.7%), and transport accidents (0.3%). In the VPA group, lung diseases (8%) and diabetes mellitus (8%) accounted for secondary causes of death. Six (2%) patients died of probable SUDEP in the PB group. The highest cause-specific SMR was found for overall brain tumor in two groups (PB group, 60.4; VPA group, 52.2), followed by cerebrovascular and heart disease. For epilepsy-related deaths (status epilepticus, 34.75 years; and probable SUDEP, 29.17 years), the mean YPLL was higher than that of other causes of death.

**Table 2:** Cause-specific proportional mortality ratio (PMR) and standardized mortality ratio (SMR) in people with convulsive epilepsy in rural northeast China.

	**Phenobarbital *N***	**PMR %**	**SMR (95% CI)**	**Sodium valproate *N***	**PMR %**	**SMR (95% CI)**
All	300	100	13.08 (11.64–14.65)	25	100	
Cerebrovascular disease	96	32	17.84 (14.45–21.78)	10	40	12.75 (6.15–23.45)
Heart disease	84	28	15.92 (12.70–19.71)	4	16	5.20 (1.42–13.32)
**Cancer**						
Tumoral (CNS excluded)	15	5	36.36 (20.35–59.97)			
Brain tumor	7	2.33	53.20 (21.39–109.60)	1	4	
Status epilepticus	12	4		1	4	
Pneumonia and lung diseases	12	4	4.47 (2.31–7.81)	2	8	
Diseases of the digestive system	7	2.33	14.21 (5.71–29.28)			
Probable SUDEP	6	2				
Diseases of the genitourinary system	4	1.33				
Suicide	3	1				
Drowning	2	0.67				
Injury	2	0.67				
Congenital Malformations	2	0.67				
Transport accidents	1	0.33				
Diabetes mellitus				2	8	
Burns				1	4	
Other diseases	4	1.33		1	4	
Unknown	43	14.34		3	12	

*CNS, central nervous system; CI, confidence interval; SUDEP, sudden unexpected death in epilepsy*.

## Discussion

This is the first large-scale epidemiological study of premature mortality in convulsive epilepsy evaluated in rural northeast China. In this study, we examined the survival analysis and the risk of premature mortality, which was assessed by MR, PMR, and SMR, and compared those between the PB and VPA groups from 2010 to 2018.

There were no major differences from the previous analyses except for the main cause of death. There were 300 and 25 decreased patients in the PB (*n* = 3,418 patients) and VPA (*n* = 498 patients) groups, respectively. The issue of disease severity is of paramount concern in studies of mortality in epilepsy (17). We are interested in whether epilepsy increases the risk of dying, and MR was considered an appropriate metric to answer this question. The overall MR (9.42%) in our study was higher than that of seven population-based studies (5.4%) (4). Compared to a study reported by Mu et al. (9) the MR of the PB group (9.96%) in our study was higher than that in rural west China (2.97% during a median follow-up duration of 28 months). High cardiovascular and cerebrovascular MRs and the longer follow-up time in our study may contribute to this difference. Ding et al. conducted two surveys for the same PB-treated cohort of PWE in 2004 (median follow-up duration of 25 months) and 2008 (median follow-up duration of 6 years), respectively. The MR in their studies increased from 1.4 to 10% in the longer follow-up (8, 10). We assumed that the longer follow-up may result in more accurate results.

Mortality risk by sex was examined for men and women in the two groups, and we observed higher SMRs among men, which was consistent with previous studies (8, 9, 19–21). In our study, men and women with epilepsy in the PB group lose ~23 and 20 years of life, respectively, which is approximately twice as much compared to that reported in a study by Gaitatzis et al. (22). Although shorter life expectancy in PWE is not surprising (23), the distinct high YPLLs in PB-treated patients in rural northeast China should be verified in additional studies. Life expectancy lost in women with epilepsy in the VPA group was higher than that in men. This finding is contrary to the results of the PB group and previous studies (22, 23). This may be limited by the small number of PWE recruited into the VPA group, or that VPA may have a greater influence on women. A higher risk of premature death in the youngest age groups was reported in most of the published studies (9, 10, 19, 23–26). Our data are consistent with those studies; the highest SMRs were in patients aged 15–19 years, and SMR decreased as the age of the patients increased. The high SMR in young adults reflects both the low mortality in the reference population and the high mortality in young people with convulsive epilepsy in rural areas (27). Since the duration of the follow-up was longer in PB than in VPA patients, we performed a survival analysis on the two cohorts, which revealed that the different treatments did not affect the MR.

We also calculated cause-specific PMR and SMR. Accurate death certificate information is critical in the study of mortality of PWE (28). However, some studies suggested that death certificates are an unreliable source of information on cause of death, and death due to epilepsy can be underestimated (29–31). Verbal autopsy, a method used increasingly in developing countries to ascertain the likely cause of death (14), was therefore adopted in our study. Epilepsy-related conditions (status epilepticus and probable SUDEP) and accidents (falls, traffic accidents, drownings, and burns) were not the leading causes of death in our study, and these findings were inconsistent with some results of previous studies (9, 19). In our study, most patients died from causes that were not strictly related to epilepsy, and the major causes of death were cerebrovascular disease and heart disease, which were also the leading causes of death of the general population in rural China in recent years (18). Previous data may explain our findings. According to the study reported by Wang et al. (32) in 2017, the prevalence of hypertension, alcohol consumption, and smoking in northeast China was higher compared to other regions of China (including west China), which may result in the highest annual incidence and mortality of stroke. Accidental death, especially drowning, was the main cause of death in western China (9, 10), but there were very few incidences of drowning in our study. This result might be explained in part by the fact that northeast China lies inland and has fewer bodies of water (e.g., ponds, paddy fields, and lakes). Mu et al. (9) estimated that the risk of drowning was 82-fold higher in their cohort than in the general population, probably because the reported area has extensive bodies of water and people often live close to water. In 2013, Ding et al. (10) confirmed that the risk was greater for those living in a waterside area than for those living in the mountains. Additionally, the public health workers in our study took advantage of every possible public channel to alert PWE and their families of the risks when a seizure occurs. PWE should avoid swimming, diving, and climbing to high places by themselves.

Several limitations need to be acknowledged. First, the absence of the number of deaths expected in the local population (northeast China) for each individual cause is a limitation of our study. Second, the VPA treatment group had a shorter duration of follow-up and smaller number than the PB group. Because PB was the first AED adopted in this epilepsy management programme, the public health workers may be more familiar with it, which affects the enrolled number in VPA group. It is thus necessary to strengthen education on AED use.

In conclusion, this is the first study on the MR and cause of death of people with convulsive epilepsy in rural northeast China. Furthermore, we compared the differences in MRs between patients treated with PB vs. VPA monotherapy. Considering the high risk of cerebrovascular disease and cardiopathy in northeast China, prevention, and local broadcasting should be improved. However, a larger cohort and longer follow-up of patients treated with VPA are needed in future studies.

## Data Availability Statement

All datasets generated for this study are included in the article/supplementary material.

## Ethics Statement

The studies involving human participants were reviewed and approved by the Ethics Committee of the First Hospital of Jilin University. Written informed consent to participate in this study was provided by the participants' legal guardian/next of kin.

## Author Contributions

DZ and GL communicated with public health workers. XZ collected the data. JL and NL analyzed and interpreted the patient data. JL wrote the manuscript. WL was a major contributors in revising the manuscript. All authors read and approved the final manuscript. All authors contributed to the article and approved the submitted version.

## Conflict of Interest

The authors declare that the research was conducted in the absence of any commercial or financial relationships that could be construed as a potential conflict of interest.
